# COVID-19 disease: CT Pneumonia Analysis prototype by using artificial intelligence, predicting the disease severity

**DOI:** 10.1186/s43055-020-00309-9

**Published:** 2020-09-25

**Authors:** Walaa Gouda, Rabab Yasin

**Affiliations:** Radiology Department, Faculty of Medicine, Menofia Univeristy, Menofia, Egypt

**Keywords:** COVID 19, Coronavirus infections, Quantitative CT analysis, Qualitative CT analysis

## Abstract

**Background:**

Since the beginning of 2020, coronavirus disease has spread widely all over the world and this required rapid adequate management; therefore, continuous searching for rapid and sensitive CT chest techniques was needed to give a hand for the clinician.

We aimed to assess the validity of computed tomography (CT) quantitative and qualitative analysis in COVID-19 pneumonia and how it can predict the disease severity on admission.

**Results:**

One hundred and twenty patients were enrolled in our study, 98 (81.7%) of them were males, and 22 (18.3%) of them were females with a mean age of 52.63 ± 12.79 years old, ranging from 28 to 83 years. Groups B and C showed significantly increased number of involved lung segments and lobes, frequencies of consolidation, crazy-paving pattern, and air bronchogram. The total lung severity score and the total score for crazy-paving and consolidation are used as severity indicators in the qualitative method and could differentiate between groups B and C and group A (90.9% sensitivity, 87.5% specificity, and 93.2% sensitivity, 87.5% specificity, respectively), while the quantitative indicators could differentiate these three groups. Using the quantitative CT indicators, the validity to differentiate different groups showed 84.1% sensitivity and 81.2% specificity for the opacity score, and 90.9% sensitivity and 81.2% specificity for the percentage of high opacity.

**Conclusion:**

Advances in CT COVID-19 pneumonia assessment provide an accurate and rapid tool for severity assessment, helping for decision-making notably for the critical cases.

## Background

Coronavirus disease 2019 (COVID-19) was firstly diagnosed in Wuhan, China; was announced by the WHO to be caused by a novel coronavirus (SARS-CoV-2); and causes a respiratory disease pandemic [1–4].

Initially, COVID-19 diagnosis was depending upon real-time reverse transcriptase polymerase chain reaction (RT RT-PCR). CT has shown to be a sensitive method for the initial evaluation of the patients [5]. On CT scan, the disease was commonly presented in the form of peripheral multifocal ground-glass opacities and consolidation [6–10].

With the dramatic increase in the patient’s number, it becomes necessary to create methods to help doctors in their war against the virus. That is why the artificial intelligence (AI) starts to share to reduce the burden on clinicians [11, 12].

So, the role of CT in the assessment of COVID-19 can be greatly optimized by the help of automated image analysis with artificial intelligence techniques allowing accurate and rapid assessment in a large number of patients, help for the fast clinical decision-making, and improve workflow efficiency. The average time for manual (semi-quantitative) CT assessment is 15 min which can be reduced into 10 s using the AI [9, 13–19].

Our study was aiming to evaluate the performance of the quantitative and qualitative CT severity scores and their usefulness as rapid and sensitive indicators for the disease severity.

## Methods

One hundred and twenty patients who were tested positive for novel coronavirus by nasopharyngeal swap were enrolled in our retrospective study in the period of 1 May and 20 June 2020. There were 98 males and 22 females with a male to female distribution of 4.5:1 and with an age range from 28 to 83 years old with mean = 52.63 ± 12.79.

The study protocol was approved by the local Ethics Committee. All patients provided a written informed consent.

Patients were stratified into three clinical groups based the WHO interim guidance [20, 21]: group A, mild cases; group B, severe cases; and group C, critical cases. Group A involves patients with mild clinical symptoms in the form of fever, mild respiratory tract manifestations, and positive CT findings of pneumonia. Group B involves patients with respiratory rate ≥ 30 times per minute, oxygen saturation ≤ 93% at rest, arterial oxygen partial pressure (PaO2)/inspired oxygen (FiO2) ≤ 300 mmHg (1 mmHg = 0.133 kPa), or significant progression of pneumonia CT findings within 24–48 h ≥ 50%. Group C involves patients that are admitted to the intensive care unit for mechanical ventilation or had a FiO2 of at least 60% or more.

### Image acquisition and analysis

All CT examination was performed using two multidetector CT scanners (Somatom Perspective, Siemens, Germany, and Optima CT 540, GE, America), using the following parameters: tube voltage = 120 kVp, tube current (regulated by automatic dose modulation), 30–75 mAs, pitch = 1–1.25 mm, matrix = 512 × 512, slice thickness = 5 mm, and FOV = 350 mm × 350 mm.

Image reconstruction was done at a slice thickness of 1–1.25 mm. All were the initial CT scans at the time of patients’ admission and are performed as non-contrast studies. Two experienced radiologists (20 years of experience) independently reviewed all the scans, and they were blinded to the patients’ clinical and laboratory data.

### Qualitative image analysis

CT severity score was estimated for each one of the five lung lobes by calculating the dissemination of the chest manifestations (opacity), namely the ground-glass opacities (GGO), consolidation, crazy-paving pattern, septal thickening, and pulmonary fibrosis giving score (0–4) for 0, 25, 50, and ≥ 75% involvement, respectively, with the sum representing the total severity scores for the whole lung (0–20).

Previous studies [3, 4] reported that the degree of consolidation and crazy-paving pattern was highly suggestive for the disease progression/peak, so we used a total sum extent of crazy-paving and consolidation as an indicator for the disease severity. The severity score for the consolidation and crazy-paving was calculated for each lobe using the same criteria (0–4 scores), and the total score for the lungs is the sum of individual lobes (0–20 scores).

### Quantitative image analysis

CT Pneumonia Analysis algorithm is designed by Siemens Healthineers to automatically identify and quantify abnormal tomographic patterns in the lungs from chest CT for research purposes. The system takes as input a non-contrasted chest CT, and identifies and 3D segments the lungs and lobes before segmenting the abnormalities. It outputs two combined measures of the severity of lung/lobe involvement, quantifying both the extent of COVID-19 abnormalities and presence of high opacities. High opacity abnormalities were shown to correlate with severe symptoms. The first disease severity measure is global, while the second is lobe-wise:

- *First global measure*
  - Percentage of opacity (PO): percentage of predicted volume of abnormalities compared to the total lung volume
  - Percentage of high opacity (PHO): percentage of predicted high opacity volume compared to the predicted volume of abnormalities
- *Second lobe-wise measure*
  - Lung severity score (LSS): the extent of abnormalities across each lobe
  - Lung high opacity score (LHOS): the extent of high opacity abnormalities for each lobe

The computed results could be used to analyze the severity and monitor the progression of abnormalities in patients exhibiting COVID-19 symptoms.

### AI-Rad Companion Research CT Pneumonia Analysis

The family of AI-powered augmented workflow solutions, running on the teamplay digital health platform, helps to reduce the burden of basic repetitive tasks and increase the diagnostic precision when interpreting medical images. Its solutions provide automatic post-processing of imaging datasets through AI-powered algorithms. The automation of routine workflows with repetitive tasks and high case volumes helps to ease the daily workflow, so that the radiologist can focus on more critical issues. This system is capable of computing the severity scores in approximately 10 s per case versus 30 min for manual annotations. These results could be used to rapidly assess the extent of lung infection and monitor the progression of abnormalities in patients exhibiting COVID-19 symptoms.

Using an artificial intelligence algorithm, the abnormal tomographic patterns commonly present in lung infections, namely ground-glass opacities (GGO) and consolidations, were automatically detected and quantified. This algorithm estimates the overall lung affection and quantifies the high opacity abnormalities using a 3D segmentation of lesions, lungs, and lobes.

Opacity score is calculated for each lobe by estimating the given region percent opacity as follows: score= 0, ≤ 25%; score = 1, 25–50%; score = 2, 50–75%; score = 3, > 75%; and score = 4 and the total score is the sum of these values.

Variable parameters are also obtained including lung volume (ml), volume of opacity (ml), percentage of opacity within a given lung region (%), volume of high opacities as absolute value (ml), a given lung region percentage of high opacities, total mean HU, given lung region mean HU of opacity, total HU standard deviation, and a given lung region opacity HU standard deviation. All these parameters are calculated for the whole lung, left lung, right lung, and per lung lobe, respectively.

### Statistical analysis of the collected data

Data were expressed in number (no.), percentage (%), mean ($\overline{x}$), and standard deviation (SD) and statistically analyzed by an IBM-compatible personal computer with SPSS statistical package version 23 (SPSS Inc. Released 2015. IBM SPSS statistics for windows, version 23.0, Armonk, NY: IBM Corp.).

ANOVA test was used for the comparison of quantitative variables between more than two groups of normally distributed data with Tukey’s test as the post hoc test while the Kruskal-Wallis test was used for the comparison of quantitative variables between more than two groups of not normally distributed data with Tamhane’s test as the post hoc test.

Pearson’s correlation was used to show correlation between two continuous normally distributed variables while Spearman’s correlation was used for not normally distributed ones.

- Chi-square test (*χ*^2^) was used to study association between qualitative variables. Whenever any of the expected cells were less than five, Fisher’s exact test was used. *Z* test was used to compare column proportions.
- Receiver operating characteristic (ROC) with respective points of maximal accuracy for sensitivity and specificity was generated to determine radiological variables’ performance. Area under the ROC curve (AUROC) measures the accuracy of the test. An area of 1 represents a perfect test; an area of 0.5 represents a worthless test. Two-sided *P* value of < 0.05 was considered statistically significant.

## Results

One hundred and twenty proven COVID 19 patients were enrolled in this retrospective including 98 (81.7%) males and 22 (18.3%) females. The patients’ age ranged from 28 to 83 years old with a mean age of 52.63 ± 12.79. There were 32 patients (26.7%) within group A, 56 patients (46.7%) in group B, and 32 patients (26.7%) in group C.

COVID-19 pneumonia CT chest manifestations generally show more common bilateral and peripheral distribution (58 patients, 48.3%) with the GGO as the commonest finding (112 patients, 93.3%) followed by the consolidation (108 patients, 90.0%) and septal thickening (66 patients, 80.0%) then the crazy-paving pattern (80 patients, 66.7%). Air bronchogram was also a common finding (108 patients, 90.0%) while pleural fibrosis and effusion were seen only in 34 patients (28.3%) and 18 patients (15.0%), respectively.

### Qualitative parameters

**Fig. 1:**
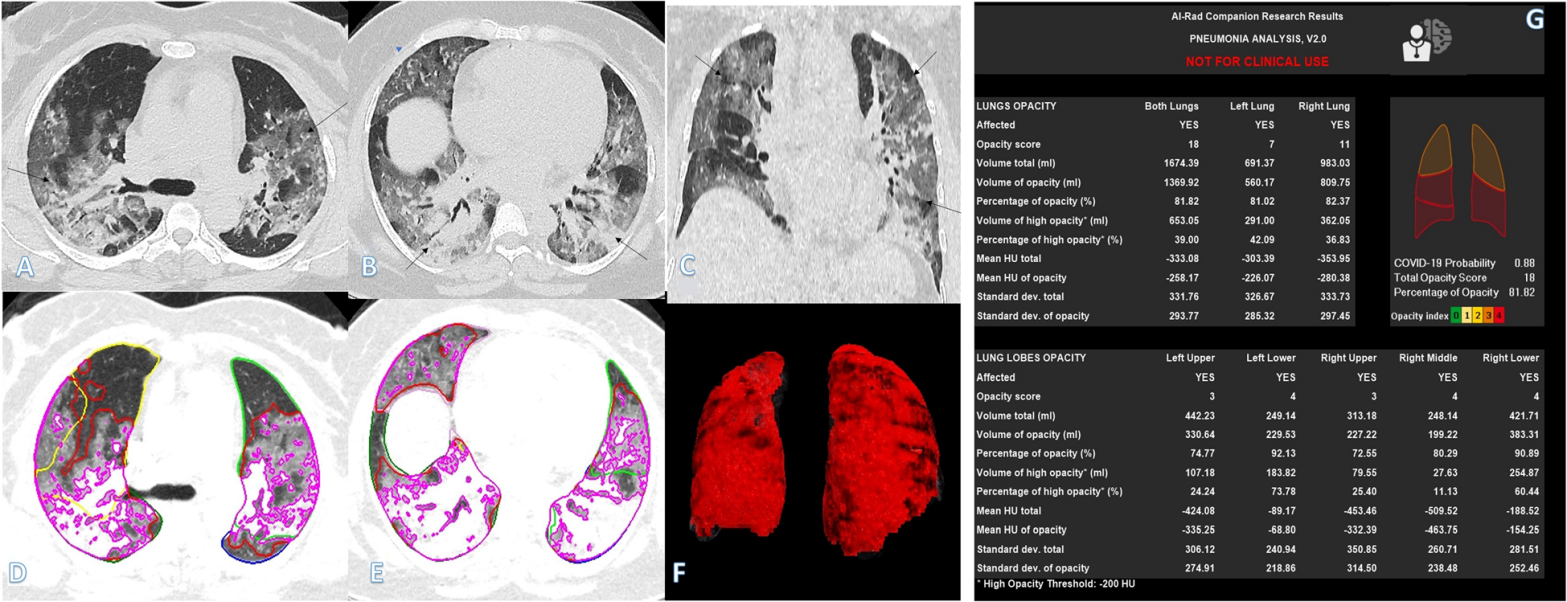
A 37-year-old female with positive COVID-19 virus. CT chest shows diffuse bilateral consolidation with air bronchogram, crazy-paving appearance, and organizing pneumonia pattern of COVID-19 (arrows in **a**–**c**). Total severity score = 18. Quantitative analysis (**d**–**g**) by AI-Rad Companion Research CT Pneumonia Analysis was presented with the measured parameters seen in table (**g**). Quantitative total opacity score was 18

Table 1 shows the comparison of the chest manifestations among different clinical groups, all chest findings: diffuse lung involvement (Fig. 1) was significantly higher in group C than other groups while peripheral and random distribution was significantly lower in group C as compared to the other groups.

**Table 1:** Comparisons of the qualitative CT findings among clinical groups

Parameters	Clinical score			*P* value	Post hoc
	A (*n* = 32), mean ± SD	B (*n* = 56), mean ± SD	C (*n* = 32), mean ± SD		
Gender				1.00	
Male	26 (81.3%)	46 (82.1%)	26 (81.3%)		
Female	6 (18.8%)	10 (17.9%)	6 (18.8%)		
Distribution				< 0.001	
Peripheral	20 (62.5%)	32 (57.1%)	6 (18.8%)^†^		
Random	10 (31.3%)	14 (25.0%)	2 (6.3%)^†^		
Diffuse	2 (6.3%)	10 (17.9%)	24 (75.0%)*		
GGO	30 (93.8%)	52 (92.9%)	30 (93.8%)	0.981	
Consolidation	22 (68.8)^†^	56 (100.0%)	30 (93.8%)	< 0.001	
Crazy-paving	8 (25.0%)^†^	42 (75.0%)^†^*	30 (93.8%)*	< 0.001	
Air bronchogram	22 (68.8%)^†^	56 (100.0%)	30 (93.8%)	< 0.001	
Septal thickening	18 (56.3%)^†^	48 (85.7%)	30 (93.8%)	< 0.001	
Pleural fibrosis	0 (0.0%)^†^	22 (39.3%)	12 (37.5%)	< 0.001	
Effusion	0 (0.0%)^†^	14 (25.0%)	4 (12.5%)	0.002	
Total severity score	5.62 ± 2.37	9.96 ± 2.64	16.12 ± 2.43	< 0.001	P1 < 0.001 P2 < 0.001 P3 < 0.001
Total score for crazy-paving and consolidation	3.00 ± 2.24	8.50 ± 2.43	14.12 ± 2.45	< 0.001	P1 < 0.001 P2 < 0.001 P3 < 0.001
No. of segments	10.37 ± 5.21	14.64 ± 3.24	18.87 ± 1.93	< 0.001	P1 < 0.001 P2 < 0.001 P3 < 0.001
No. of lobes	4.06 ± 1.31	4.82 ± 0.60	4.93 ± 0.24	< 0.001	P1 0.011 P2 0.002 P3 0.508

*P1* mild vs moderate, *P2* mild vs severe, *P3* moderate vs severe

^†^Significantly lower than other groups in the same category

*Significantly higher than other groups in the same category

Consolidation, air bronchogram, septal thickening, lung fibrosis, and pleural effusion have a significant difference between group A and other groups (B and C) with *P* value < 0.001, so it can differentiate between them. But it could not differentiate between groups B and C.

**Fig. 2:**
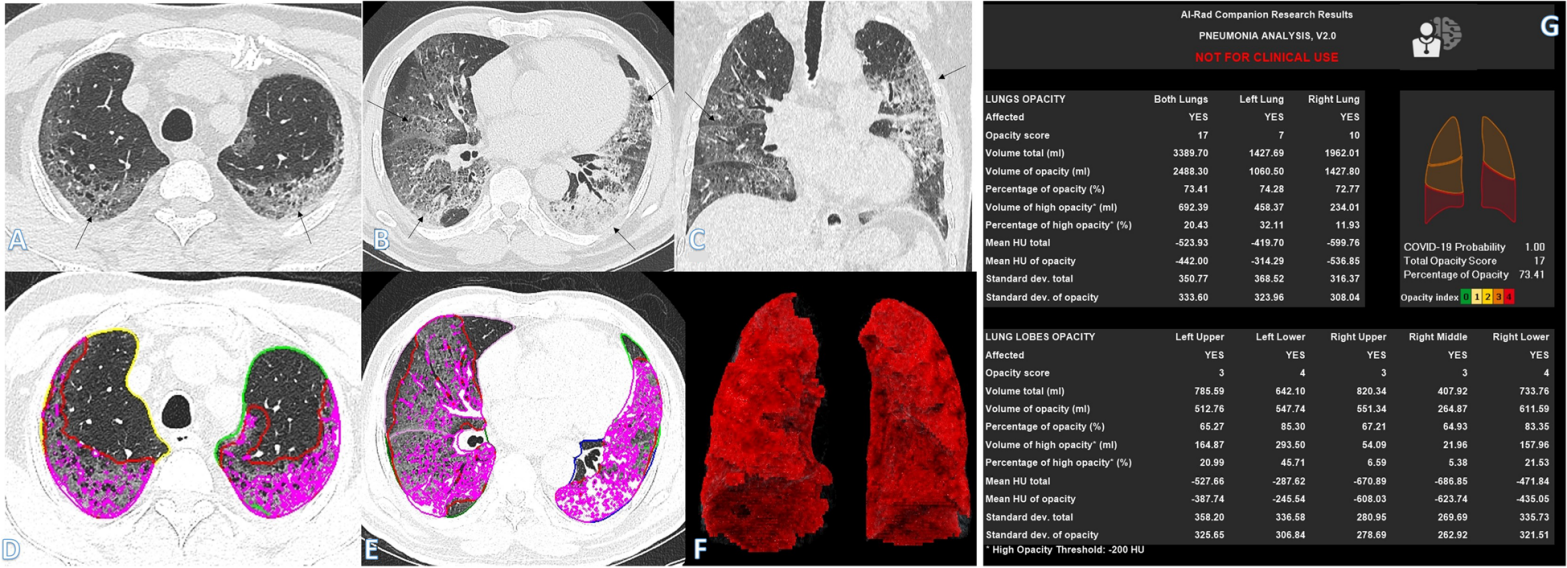
A 64-year-old male with positive COVID-19 virus. CT chest shows centrilobular emphysematous changes with bilateral diffuse ground-glass opacities with crazy-paving appearance with more involvement of both lower lobes (arrows in **a**–**c**). Total severity score = 17. Quantitative analysis by AI-Rad Companion Research CT Pneumonia Analysis was presented (**d**–**g**) with the measured parameters seen in table (**g**). Quantitative total opacity score was 17

Crazy-paving pattern (Fig. 2) was significantly higher in group C than other groups (A and B) and significantly lower in group A than other groups (B and C). It was the only chest finding that could differentiate between all groups with *P* value < 0.001.

GGO showed similar distribution in different groups with no statistically significant difference between them.

There was a highly statistical significance between the different groups as regards the calculated total severity score and total score for crazy-paving and consolidation as well as number of involved lung segments with *P* value < 0.001, while for the number of involved lobes of the lungs, there was a statistical significance between group A and other groups (B and C) with no statistical significance between group B and group C.

Groups B and C also showed longer time interval between the disease onset and the initial CT scan than group A, yet with no significant difference between the two groups (B and C).

### Quantitative indicators

**Fig. 3:**
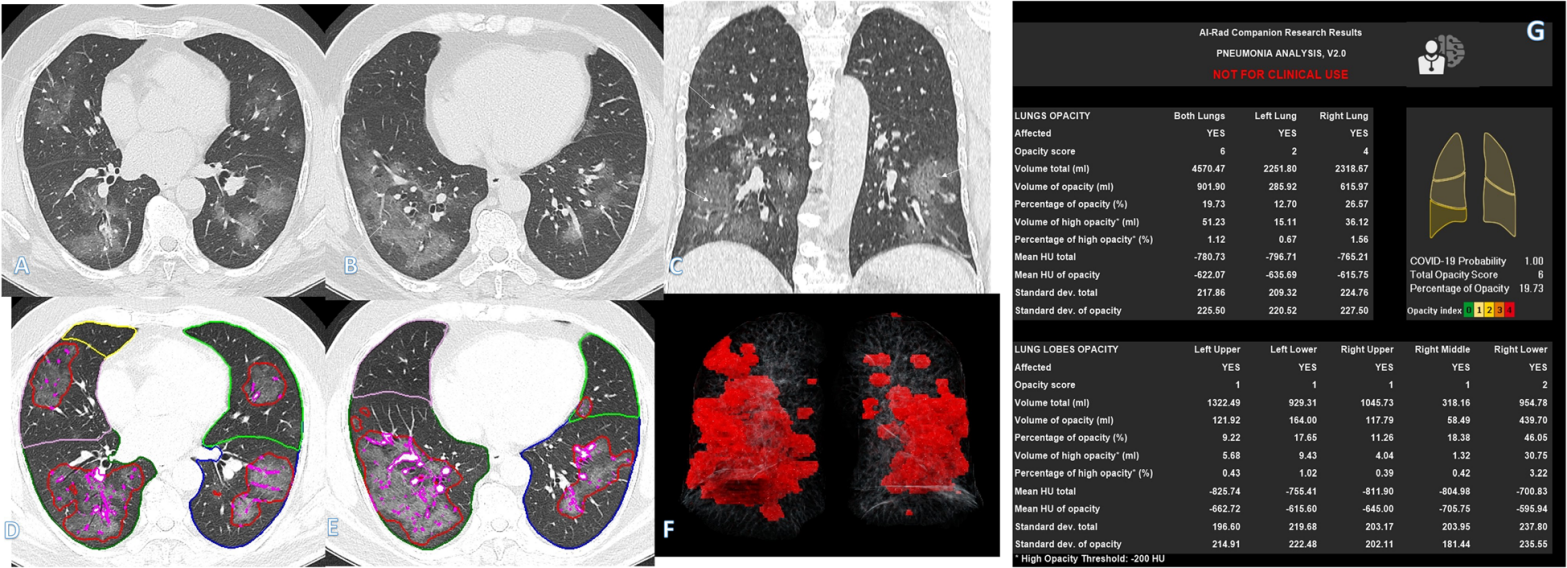
A 61-year-old male with positive COVID-19 virus. CT chest shows bilateral scattered random ground-glass opacities (arrows) with more involvement of the right lower lobe (arrows in **a**–**c**). Total severity score = 7. Quantitative analysis by AI-Rad Companion Research CT Pneumonia Analysis was presented (**d**–**g**) with the measured parameters seen in table (**g**). Quantitative total opacity score was 6

**Fig. 4:**
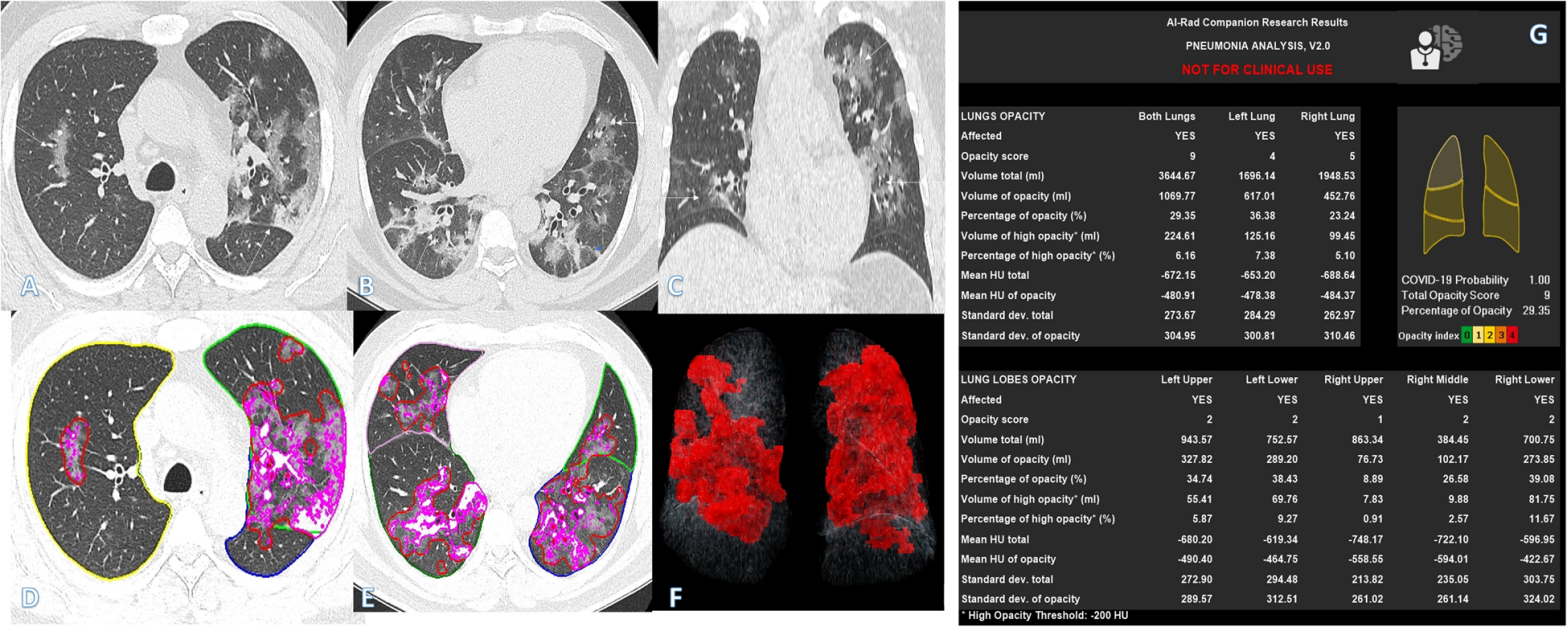
A 54-year-old male with positive COVID-19 virus. CT chest shows bilateral scattered random ground-glass opacities and consolidation patches with air bronchogram (arrows) with more involvement of the left upper and both lower lobes (arrows in **a**–**c**). Total severity score = 8. Quantitative analysis by AI-Rad Companion Research CT Pneumonia Analysis was presented (**d**–**g**) with the measured parameters seen in table (**g**). Quantitative total opacity score was 9

**Fig. 5:**
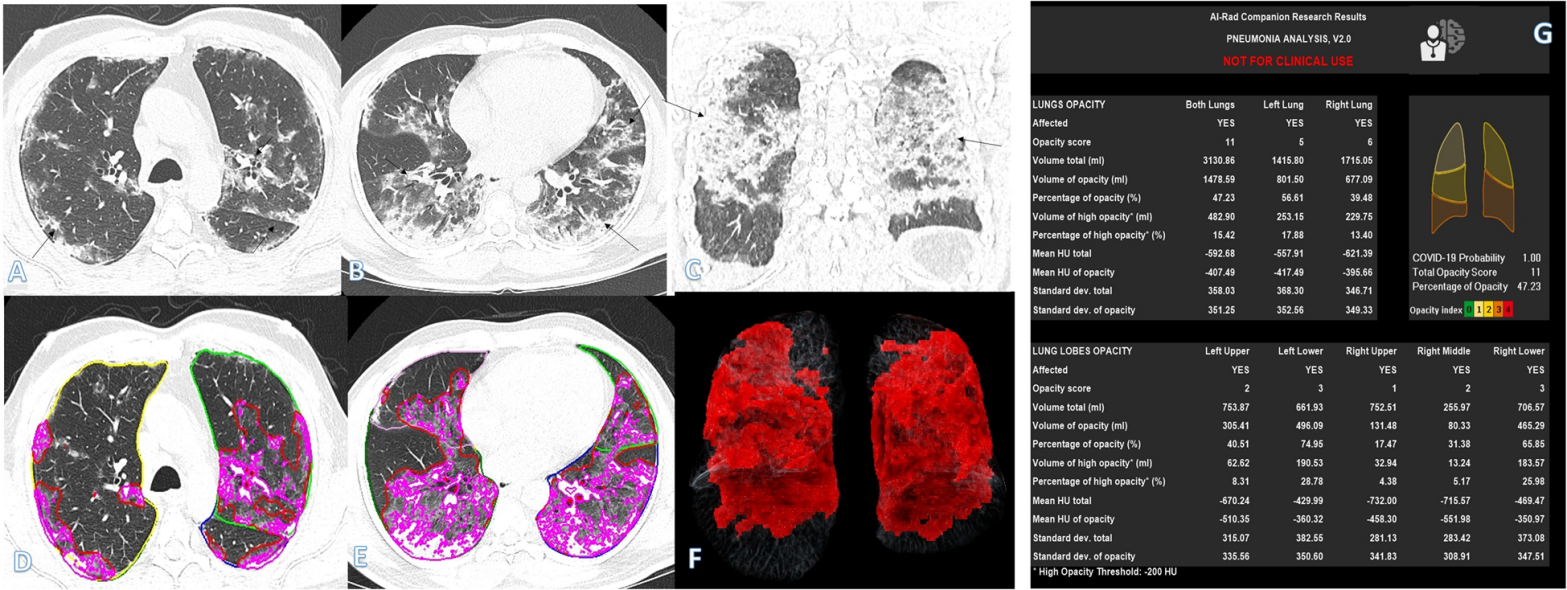
A 54-year-old male with positive COVID-19 virus. CT chest shows bilateral consolidation patches with air bronchogram, crazy-paving appearance mainly peripheral and basal, and subpleural lines (arrows) with more involvement of the left upper and both lower lobes (arrows in **a**–**c**). Total severity score = 11. Quantitative analysis (**d**–**g**) by AI-Rad Companion Research CT Pneumonia Analysis was presented with the measured parameters seen in table (**g**). Quantitative total opacity score was 11

**Fig. 6:**
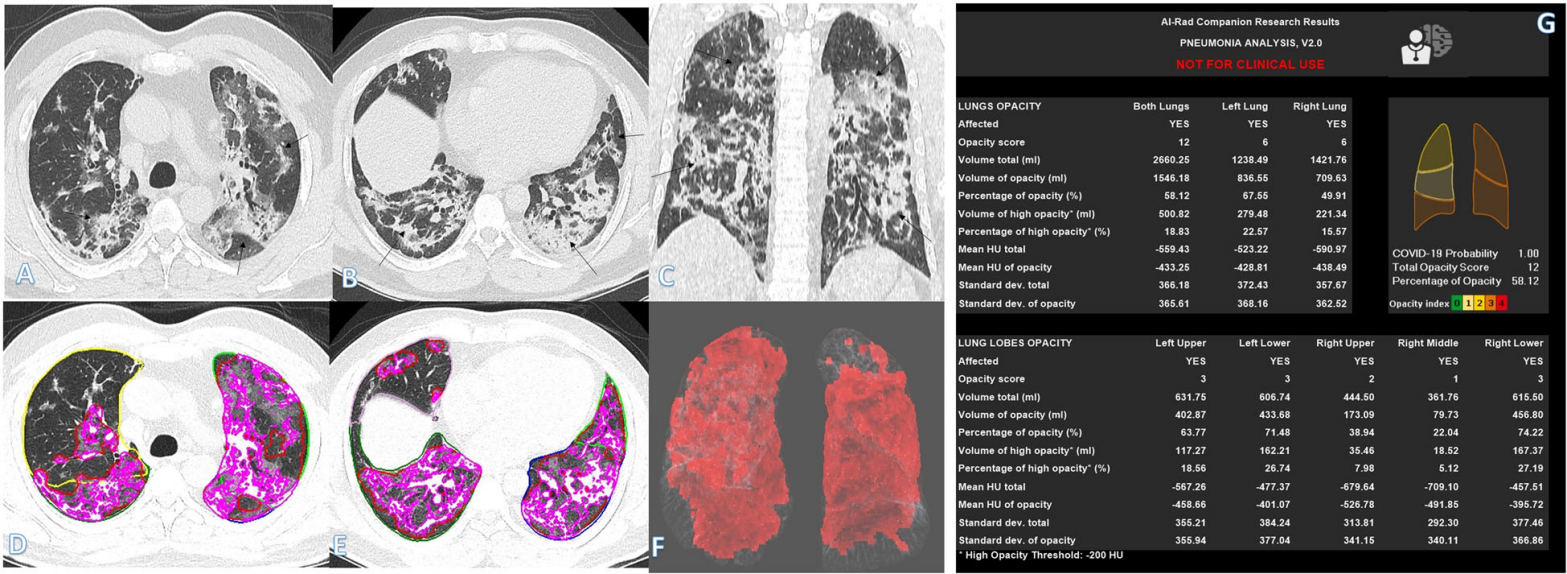
A 46-year-old male with positive COVID-19 virus. CT chest shows diffuse bilateral confluent consolidation with air bronchogram, crazy-paving appearance, subpleural thickening with fibrosis, and organizing pneumonia pattern of COVID-19 (arrows in **a**–**c**). Total severity score = 13. Quantitative analysis (**d**–**g**) by AI-Rad Companion Research CT Pneumonia Analysis was presented with the measured parameters seen in table (**g**). Quantitative total opacity score was 12

As regards the quantitative analysis, most of its parameters were significantly different among different clinical groups. The total opacity score, percentage of opacity, volume of opacity, and MLD were significantly higher in groups B and C compared to group A as well as between group B and C (all *P* value < 0.001) (Table 2). The total lung volume was significantly lower in group C compared to group A (Figs. 1, 2, 3, 4, 5, and 6).

**Table 2:** Quantitative parameters at different clinical groups

Parameters	Clinical score			*P* value	Post hoc
	A (*n* = 32), mean ± SD	B (*n* = 56), mean ± SD	C (*n* = 32), mean ± SD		
Total opacity score	5.81 ± 2.64	10.92 ± 3.77	17.00 ± 2.59	< 0.001	P1 < 0.001 P2 < 0.001 P3 < 0.001
Lung volume	3512.89 ± 1165	2874.89 ± 873.80	2257.30 ± 608.25	< 0.001	P1 0.028 P2 < 0.001 P3 0.001
LAV	− 611.34 ± 156.72	− 538.67 ± 80.33	− 483.62 ± 74.87	< 0.001	P1 0.056 P2 < 0.001 P3 0.006
HAV	− 396.44 ± 183.77	− 311.10 ± 89.04	− 260.98 ± 67.69	< 0.001	P1 0.053 P2 0.012 P3 < 0.001
Percentage of opacity	14.73 ± 12.83	40.51 ± 21.19	73.75 ± 15.75	< 0.001	P1 < 0.001 P2 < 0.001 P3 < 0.001
Volume of opacity	460.38 ± 331.83	1079.76 ± 469.84	1681.01 ± 562.28	< 0.001	P1 < 0.001 P2 < 0.001 P3 < 0.001
Volume of high opacity	98.40 ± 79.74	314.84 ± 124.39	564.70 ± 192.39	< 0.001	P1 < 0.001 P2 < 0.001 P3 < 0.001
Percentage of high opacity	3.30 ± 3.14	12.32 ± 7.47	25.52 ± 7.50	< 0.001	P1 < 0.001 P2 < 0.001 P3 < 0.001
Mean HU total (MLD)	− 710.59 ± 91.50	− 593.10 ± 93.75	− 435.77 ± 88.19	< 0.001	P1 < 0.001 P2 < 0.001 P3 < 0.001
Mean HU of opacity	− 489.42 ± 120.72	− 386.66 ± 86.71	− 352.08 ± 60.09	< 0.001	P1 < 0.001 P2 < 0.001 P3 0.089

*P1* mild vs moderate, *P2* mild vs severe, *P3* moderate vs severe, *HAV* high attenuation value, *LAV* low attenuation value, *MLD* mean lung density

LAV and HAV could not differentiate between group A and group B, but it showed a high statistically significant difference between groups A and C for LAV (*P* value < 0.001), and high significant difference between groups B and C for HAV (*P* value < 0.001). All the other quantitative parameters showed significant difference between different groups except the mean HU of the opacity that showed no significant difference between both groups B and C (*P* value = 0.089).

### Clinical group stratification in relation to CT parameters

The cutoff values of statistically significant CT parameters were estimated by applying the curves of the receiver operating characteristic to assess the sensitivity and the specificity of these indicators to differentiate between group A from the other two groups (Table 3). We found that the cutoff values to differentiate between group A from other groups were 7.5 for total severity score 7.5 with 90.9% sensitivity and 87.5% specificity, while 8.5 for total opacity score with 84.1% sensitivity and 81.2% specificity. Total score for crazy-paving and consolidation > 5.5 can differentiate between group A from other groups (B and C) with 93.2% sensitivity and 87.5% specificity. LAV and HAV had low specificity to differentiate between groups 68.7% and 62.5%, respectively. When more than 12 lung segments are involved, it can differentiate group A from other groups with 79.5% sensitivity and 43.7% specificity.

**Table 3:** Validity of different parameters in relation to the clinical grouping (mild vs moderate and severe)

Parameters	AUC	Cutoff	Sensitivity (%)	Specificity (%)
Total severity score	0.947	7.50	90.9	87.5
Total score for crazy-paving and consolidation	0.979	5.50	93.2	87.5
Total opacity score	0.920	8.50	84.1	81.2
LAV	0.709	− 573.71	75.0	68.7
HAV	0.729	− 392.53	81.8	62.5
Percentage of opacity	0.908	23.81	86.0	81.2
Percentage of high opacity	0.947	5.61	90.9	81.2
No. of segments affected	0.810	12.50	79.5	43.7
Mean HU total (MLD)	0.876	− 637.70	81.8	81.9
Lung volume	0.723	3135.20	77.3	56.2

*HAV* high attenuation value, *LAV* low attenuation value, *MLD* mean lung density

The cutoff value for MLD was − 637.7 to differentiate between different groups with 81.8% sensitivity and 81.9% specificity.

**Fig 7:**
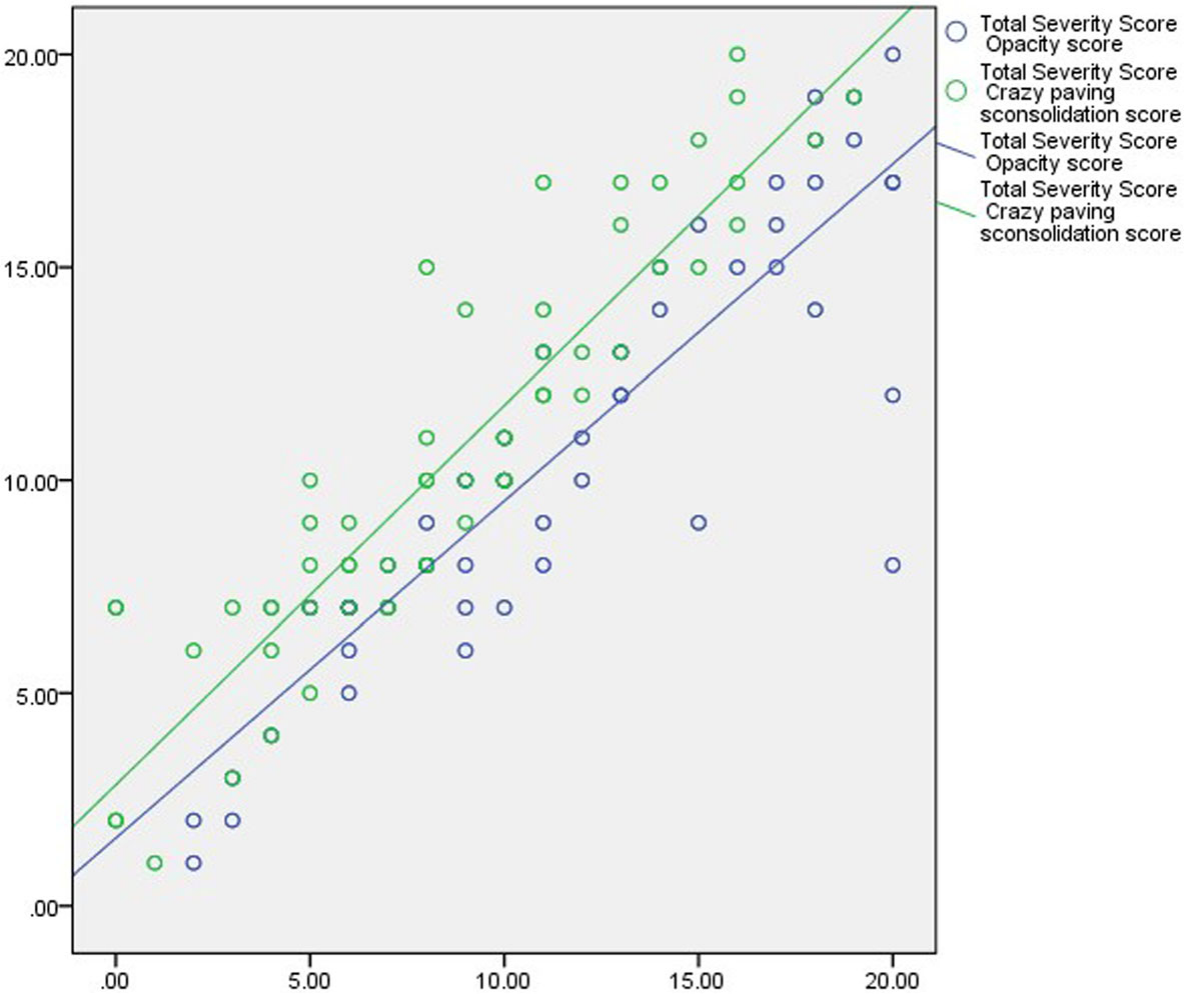
Scatter plot of the total severity score with the opacity score and crazy-paving consolidation score

There was a high statistical significance between total severity score, total opacity score, and total score for crazy-paving and consolidation in relation to the clinical grouping with *P* value < 0.001 (Table 4 and Fig. 7).

**Table 4:** Spearman’s correlation between total severity score, total opacity score, and total score for crazy-paving and consolidation in relation to clinical grouping

Scores	Clinical grouping		Total severity sore	
	*r*	*P* value	*r*	*P* value
Total severity score	0.850	< 0.001	–	–
Total opacity score	0.788	< 0.001	0.895	< 0.001
Total score for crazy-paving and consolidation	0.880	< 0.001	0.927	< 0.001

So, most of the qualitative parameters could not differentiate group C from group B while quantitative parameters provide an easy, rapid, and highly sensitive tool for accurate differentiation between the different clinical groups.

## Discussion

COVID-19 disease is a highly contagious illness that showed rapid worldwide spread. Early disease diagnosis is very crucial for disease containment strategies and the management of the patients [1, 2]. The significant increase of the patients’ number creates a great challenge for COVID-19 laboratory testing owing to the limited facilities and inadequate supply of nucleic acid kits. Using chest radiographs at the initial disease assessment showed a significant number of false negatives due to its deficiency to detect the early disease abnormalities [3].

Chest CT shows significantly higher sensitivity for detection of mild pulmonary manifestations in early disease stages. That is why, chest CT has become a forefront diagnostic tool during the COVID-19 pandemic [4].

Previous literature has shown similar results [8–11], concerning the predominance pattern of abnormal chest CT manifestations to be bilateral and peripheral with the GGO and consolidation which are the commonest findings; however, they are not indictors for different clinical group stratifications. Correlation with pathological findings can give explanation for this, as in early disease stages virus invasion causes alveolar damage with interstitial pulmonary infiltration manifested as alveolar edema with protein exudate and interlobular thickening of the interstitium. Through the disease progression, diffuse alveolar damage with cellular fibromyxoid exudate can induce critical disease stage, yet both types of exudate manifest as GGO [5, 6].

Disease progression (severe/critical stages) is probably induced by more infiltration of the lung parenchyma and interstitium [7, 8] which is caused by invasion of the respiratory epithelium by the virus with disseminated damage of the alveoli, necrotizing bronchitis, and total alveolar filling by the inflammatory exudate. This explains the increase of consolidation and crazy-paving pattern frequencies in severe/critical cases comparing to the mild ones [5, 6].

In our study, qualitative chest findings such as consolidation, air bronchogram, septal thickening, lung fibrosis, and pleural effusion showed a significant difference between group A and other groups (B and C) with *P* value < 0.001, but it could not differentiate between groups B and C.

This agreed with Lyu et al. who stated that using qualitative indicators could not differentiate group C from group B, but quantitative indicators could distinguish them [3].

In our study, crazy-paving pattern could differentiate between all groups with high statistical significance (*P* value < 0.001). It was significantly higher in group C than other groups (A and B) and significantly lower in group A than other groups (B and C). The total score for crazy-paving and consolidation > 5.5 could differentiate between group A from other groups (B and C) with 93.2% sensitivity and 87.5% specificity.

This agreed with Lyu et al. who had proposed the use of the total score for crazy-paving and consolidation as indicator for differentiation of different clinical groups and proved its significance; higher total score for crazy-paving and consolidation > 4 had 87% sensitivity and 44% specificity [3].

Our study showed that the number of involved lung segments and lobes was significantly higher at different disease stages with positive correlation with the disease progression. The whole lung total severity score has been shown as a poor prognostic indicator in patients with COVID-19. We found that the cutoff values to differentiate between group A from other groups were 7.5 for total severity score 7.5 with 90.9% sensitivity and 87.5% specificity. Involvement of more than 12 lung segments could differentiate group A from other groups with 79.5% sensitivity and 43.7% specificity.

Lyu et al. found that in severe case, the number of involved segments increased with statistically significant difference (> 8, sensitivity and specificity of 100% and 37%) [3].

Li et al. assessed the total severity score of COVID-19 patients, and they found that TSS for diagnosing severe-critical type was 0.918. The TSS cutoff of 7.5 had 82.6% sensitivity and 100% specificity [7], while Lyu et al. described TSS > 10 had 67% sensitivity and 74% specificity [3].

Also, Chung et al. did a study on 21 cases of COVID-19 and found the total lung severity score ranged from 0 (in the three normal CT examinations) to a maximum of 19, with a mean score of 9.9. The patient with the highest lung severity score was admitted to the intensive care unit [22].

The time interval between the initial CT scan and the disease onset was significantly longer in severe/critical cases compared to mild ones, and this might be partly due to that some cases were only hospitalized with progression of the disease symptoms.

Comparing the quantitative parameters among different clinical groups, most of them were significantly different in our study. The total opacity score, percentage of opacity, and volume of opacity were significantly higher in severe cases compared to the non-severe cases, and these were consistent with previous results [4, 7].

Different from previous studies of the quantitative analysis [4, 11, 17] that assessed the disease extension depending on the quantifying opacification percentage, our study also envaulted the relative volume of normal lung density which was significantly lower in critical cases and this could be very helpful for the management of these patients and add an important value of quantitative analysis in clinical practice.

The cutoff value for MLD in our study was − 637.7 to differentiate between different groups with 81.8% sensitivity and 81.9% specificity. Lyu et al. found in their study that critical cases showed higher MLD > − 779 HU with sensitivity and specificity of 100% and 73% [3].

We observed in our data that the HAV and percentage of high opacity were significantly higher in severe cases denoting high-density lesions, which match with the increased total score for crazy-paving and consolidation in the qualitative method. HAV > − 392.53 and the percentage of high opacity > 5.61 showed 81.8% and 90.9% sensitivity and 62.5% and 81.2% specificity for the detection of critical/severe cases. MLD at cutoff value > − 637.7 HU showed 81.8% sensitivity and 81.9% specificity for clinical group stratification.

This agreed with Lyu et al. who stated that HAV values increased in more severe cases due to increased high-density lesions. The higher HAV values (above than − 200 HU) are seen in the critical cases [3].

The quantitative pneumonia analysis was standardized depending upon the changes in lung density and volume changes, except for cases with co-existent chest condition where the manual adjustment was performed if necessary, to ensure the accuracy of lung segmentation.

The limitations of our study included a specific software that is required for the quantitative CT application which may restrict its clinical application. That is why the qualitative analysis can give a hand for initial disease assessment as it also showed a good sensitivity and specificity for disease stratification allowing early management of the critical cases. Our study included only the initial CT study; assessment of the follow-up scans may be recommended in later researches.

## Conclusion

We concluded that the qualitative parameters including the whole lung total severity score and the total score of crazy-paving and consolidation can be used as a good indicator for disease stratification, while the other parameters could not distinguish moderate and severe disease stages. Quantitative parameters have been shown to be helpful in this and provide accurate discrimination of this intermediate stage from severe one.

## Data Availability

All data and material are available.

## Abbreviations

CT: Computed tomography
SARS: Severe acute respiratory syndrome
RT RT-PCR: Real-time reverse transcriptase polymerase chain reaction
AI: Artificial intelligence
GGO: Ground-glass opacities
HU: Hounsfield unit
MLD: Mean lung density
LAV: Low attenuation value
HAV: High attenuation value
TSS: Total severity score
WHO: World Health Organization
SD: Standard deviation
ROC: Receiver operating characteristic
AUROC: Area under the ROC curve
PO: Percentage of opacity
PHO: Percentage of high opacity
LSS: Lung severity score
LHOS: Lung high opacity score
